# Dark-Induced Barley Leaf Senescence – A Crop System for Studying Senescence and Autophagy Mechanisms

**DOI:** 10.3389/fpls.2021.635619

**Published:** 2021-03-15

**Authors:** Ewelina Paluch-Lubawa, Ewelina Stolarska, Ewa Sobieszczuk-Nowicka

**Affiliations:** Department of Plant Physiology, Faculty of Biology, Adam Mickiewicz University in Poznań, Poznań, Poland

**Keywords:** autophagy, cell death, cell survival, developmental senescence, senescence model, sources and sinks communication, stress-induced senescence

## Abstract

This review synthesizes knowledge on dark-induced barley, attached, leaf senescence (DILS) as a model and discusses the possibility of using this crop system for studying senescence and autophagy mechanisms. It addresses the recent progress made in our understanding of DILS. The following aspects are discussed: the importance of chloroplasts as early targets of DILS, the role of Rubisco as the largest repository of recoverable nitrogen in leaves senescing in darkness, morphological changes of these leaves other than those described for chloroplasts and metabolic modifications associated with them, DILS versus developmental leaf senescence transcriptomic differences, and finally the observation that in DILS autophagy participates in the circulation of cell components and acts as a quality control mechanism during senescence. Despite the progression of macroautophagy, the symptoms of degradation can be reversed. In the review, the question also arises how plant cells regulate stress-induced senescence via autophagy and how the function of autophagy switches between cell survival and cell death.

## Introduction

In plants, senescence is a highly controlled and active process requiring global metabolic reprogramming, aimed at organized disintegration and remobilization of valuable resources (Himelblau and Amasino, 2001; Maillard et al., 2015). It is a fundamental aspect of plant development, necessary to optimize resource allocation and promote phenotypic plasticity to adapt to the environment under restricted conditions. Being photoautotrophic, plants rely mainly on their leaves to support their growth. Leaves are organs optimized for the use of light energy and the subsequent production of photosynthates while minimizing total anabolic cost. In terms of stress conditions, this can be beneficial to the plant if a leaf which is not photosynthetically productive undergoes senescence, thus making its resources available to other organs. Therefore, the induction of senescence must be strictly controlled to avoid unnecessary activation only under temporarily unfavorable conditions. In congruence with the importance of leaves in photosynthesis, light plays an essential role in regulating leaf senescence. For many species of plants, a lack of light in the form of strong shading or darkening of the leaves leads to rapid senescence, especially when only parts of the plant are affected (reviewed in Liebsch and Keech, 2016).

Dark-induced senescence has been used experimentally as an easy way to study the progress of leaf senescence. However, detailed studies of gene expression patterns have revealed discrepancies between the dark-induced and developmentally controlled process (Becker and Apel, 1993; Lee et al., 2001; Buchanan-Wollaston et al., 2005; van der Graaff et al., 2006; Guo and Gan, 2012; Roberts et al., 2017). The relevance of investigations on dark-induced senescence has often been discussed, but shade is an important scenario for crop yields in dense canopies. Under field conditions, crops are likely to benefit from the lower leaves of the canopy undergoing senescence and thus re-mobilizing nutrients for use in the upper, photosynthetic parts of the plants. Dark-induced leaf senescence (DILS) results in a clear loss of chlorophyll, disassembly of cellular elements and a lack of photosynthetic activity, none of which can be distinguished from the age-dependent natural senescence (Buchanan-Wollaston et al., 2003, 2005). However, the lack of coordinated cell development within a single leaf introduces complexity in the leaf senescence study. Thus, induced senescence, which directs a synchronous process, like dark-induced senescence, has become relevant (Kleber-Janke and Krupinska, 1997; Gepstein et al., 2003; Lin and Wu, 2004; Feller et al., 2008; Christiansen and Gregersen, 2014; Sobieszczuk-Nowicka et al., 2015, 2016; Law et al., 2018). It also eliminates misleading factors that coincide with developmental senescence, such as bolting or flowering (Gregersen et al., 2008). Widely used experimental setups to study dark-induced senescence in barley are (i) detached leaf in darkness (e.g., Becker and Apel, 1993; Legocka and Zajchert, 1999; Chrost and Krupinska, 2000; Rosiak-Figielek and Jackowski, 2000; Chrost et al., 2004; Żelisko and Jackowski, 2004; Conrad et al., 2007; Sobieszczuk-Nowicka et al., 2009, 2015; Kucharewicz et al., 2017; Janečková et al., 2019) (ii) whole plant in darkness (e.g., Kleber-Janke and Krupinska, 1997; Krause et al., 1998; Roulin et al., 2002; Simova-Stoilova et al., 2002; Arnao and Hernández-Ruiz, 2009; Jajić et al., 2014; Avila-Ospina et al., 2015; Zmienko et al., 2015; Sobieszczuk-Nowicka et al., 2016, 2018), and (iii) individually darkened attached leaf, whilst the rest of the plant remained in a normal photoperiod condition (e.g., Rolny et al., 2011; Shi et al., 2012; Christiansen et al., 2016). As the course of the senescence process is related to plant species, plant developmental stage, and plant environmental conditions these treatments cannot be considered the same. However, darkness induces some series of transformations at the cytological, biochemical and molecular levels common within these setups. These features are summarized in Table 1 and are discussed in the context of the transformations that occur in the DILS program (Sobieszczuk-Nowicka et al., 2018). DILS program setup are barley seedlings grown in growth chamber for 7 days under controlled conditions (day/night 16/8 h, 23°C, light intensity 150 μmol m^–2^ s^–1^, 60% humidity). Pots with seedlings on seventh day of growth are transferred to dark conditions to initiate senescence.

**TABLE 1:** Overview of the experimental setups of dark-induced barley leaf senescence assays employed through the different studies cited in this review, and comparison of assessed parameters against DILS (Sobieszczuk-Nowicka et al., 2018).

**Reference^1^**	**Experimental setup^2^**	**Parameters^3^**	**Assessed parameter in:**	
			**Reference Paper**	**DILS^4^**
Christiansen et al. (2016)	21-day-old, 2nd leaf, IDL, 3D	Chlorophyll	Decreased relative chlorophyll content	Decreased chlorophyll autofluorescence and chlorophyll content
Kleber-Janke and Krupinska (1997)	9-day-old, 1st leaf, WDP, 2D	Chlorophyll	Decreased chlorophyll content	Decreased chlorophyll autofluorescence and chlorophyll content
		Maximum quantum yield of PSII of the dark-adapted state (Fv/Fm)	Decreased Fv/Fm	Decreased Fv/Fm
		Rubisco	Reduced level of Rubisco large subunit transcript	Reduction in Rubisco large subunit transcript and protein levels
Krause et al. (1998)	9-day-old, 1st leaf, WDP, 2D	Rubisco	Reduced level of Rubisco large subunit transcript	Reduction in Rubisco large subunit transcript and protein levels
Lichtenthaler and Grumbach (1974)	6-day-old, 1st leaf + coleoptile, WDP, 4D	Chlorophyll	Decreased chlorophyll content	Decreased chlorophyll autofluorescence and chlorophyll content
		Thylakoid system	Degradation of thylakoids determined by the destruction of prenyl lipids	Degradation of thylakoids observed in leaf tissue ultrastructure
Peterson and Huffaker (1975)	7-day-old, 1st leaf, DET, 3D	Chlorophyll	Decreased chlorophyll content	Decreased chlorophyll autofluorescence and chlorophyll content
		Rubisco	Reduced level of Rubisco protein	Reduction in Rubisco large subunit transcript and protein levels
Roberts et al. (2017)	15-day-old, 3rd leaf, DET, 6D	Chlorophyll	Decreased relative chlorophyll content	Decreased chlorophyll autofluorescence and chlorophyll content
		Subtilases	Increased level of subtilases transcript	Decreased level of subtilases transcript
Rolny et al. (2011)	13-day-old, 1st leaf, DET, IDL, 8D	Chlorophyll	Decreased relative chlorophyll content	Decreased chlorophyll autofluorescence and chlorophyll content
Scharrenberg et al. (2003)	9-day-old, 1st leaf, WDP, 6D	Chlorophyll	Decreased relative chlorophyll content	Decreased chlorophyll autofluorescence and chlorophyll content
		Maximum quantum yield of PSII of the dark-adapted state (Fv/Fm)	Decreased Fv/Fm	Decreased Fv/Fm
		Level of cysteine protease transcript	Increased level of cysteine protease transcript	Increased level of cysteine protease transcript
Scheumann et al. (1999)	7-day-old, 1st leaf, DET, 8D	Chlorophyll	Decreased chlorophyll content	Decreased chlorophyll autofluoresence and chlorophyll content
Simova-Stoilova et al. (2002)	10-day-old, 1st leaf, WDP, 5D	Rubisco	Decreased level of Rubisco protein	Reduction in Rubisco large subunit transcript and protein levels
Spundova et al. (2003)	Plants in growth phase 1.2 according to Feekes (1941), 1st leaf, DET, 5D	Chlorophyll	Decreased chlorophyll content	Decreased chlorophyll autofluorescence and chlorophyll content
		Effective quantum yield of PSII electron transport (ΦPSII)	Decreased ΦPSII	Decreased ΦPSII
		Thylakoid system	Degradation of thylakoids observed in leaf tissue ultrastructure	Degradation of thylakoids observed in leaf tissue ultrastructure
		Number of plastoglobuli	Increased number of plastoglobules observed in leaf tissue ultrastructure	Increased number of plastoglobules observed in leaf tissue ultrastructure
Wood et al. (1998)	7-day-old, 2nd leaf, WDP, 5D	Single strand nucleases (SSN)	Increased SSN enzyme activity	Increased level of SSN transcript

*^1^Literature references to dark-induced barley leaf senescence as given in the main text. ^2^Experimental setups used in different studies of senescence analysis: age at which the leaf was investigated, type of dark induction (DET, detached leaf in darkness; EDP, whole plant in darkness; IDL, individually darkened attached leaf), the number of days in darkness (D) and days of light re-exposure (L). ^3^The parameters that were used in the study of DILS models (Sobieszczuk-Nowicka et al., 2018). ^4^Barley (*Hordeum vulgare*) seedlings were grown in growth chamber for 7 days under controlled conditions (day/night 16/8 h, 23°C, light intensity 150 μmol m^–2^ s^–1^, 60% humidity). Thereafter plants in growth phase 11 according to BBCH scale (Lancashire et al., 1991), were transferred to darkness for 3/7/10D. For experiments that determined the optimal day(s) of darkness when the physiological recovery from senescence becomes irreversible, plants grew in sequence: 3D+2/5/7L, 7D+2/5/7L, and 10D+2/5/7L.*

The genome resources available for *Arabidopsis* have made it a very attractive model of identification and functional analysis of genes regulated by senescence (Buchanan-Wollaston et al., 2003, 2005; Breeze et al., 2011). However, in many plants, such as barley, the removal of developing flowers and pods significantly extends the life of their leaves, while in *Arabidopsis*, male-sterile mutants or plants from which developing bolts have been removed do not extend the life of leaves. Because of these differences, cereal leaves must be used as an equivalent to the *Arabidopsis* model for leaf senescence studies in cereal (*Zea mays* – Smart et al., 1995; *Oryza sativa* – Lee et al., 2001; *Triticum aestivum* – Uauy et al., 2006; and *Hordeum vulgare* – Kleber-Janke and Krupinska, 1997; Jukanti et al., 2008; Christiansen and Gregersen, 2014; Avila-Ospina et al., 2015; Springer et al., 2015; Wehner et al., 2015; Sobieszczuk-Nowicka et al., 2018). Clear differences in the senescence program of *Arabidopsis* compared to monocotyledonous plants were found. The senescence in cereals is generally regulated at the single leaf level. Nutrients from older leaves are remobilized for younger leaves and ultimately for the flag leaf, thus contributing to the nutrients necessary for the development of the grain. Cereal leaves have a meristem base, the leaf tip consists of older cells, and younger ones are concentrated at the base of the leaf. This cell organization makes it easier to differentiate the progression of senescence (Gregersen et al., 2008).

Understanding both dark- and shade-induced senescence is of great economic importance as it can significantly shorten the shelf life after harvest and lead to significant crop losses (Gan and Amasino, 1997; Nam, 1997; Buchanan-Wollaston et al., 2003; Liu et al., 2015; Schippers et al., 2015). Significant progress has been made in our understanding of leaf senescence and its basic regulation at the molecular level over the last decades. Furthermore, a theoretical model (senescence window concept) has emerged which explains how senescence competence is determined during leaf development and how internal and external factors are integrated with age to determine the duration of senescence (Jing et al., 2002). Also, much of the fundamental knowledge about senescence regulation has been tested in cultivated plant species for its potential use to improve productivity. This includes stay-green features (Thomas and Ougham, 2014) and pSAG12:IPT technology (Gan and Amasino, 1995). Further clarification of the senescence window concept and a change that gives plants competence for senescence will allow for more targeted strategies to manipulate senescence by focusing on the different phases of development. Many researchers have discovered that it is extremely difficult to try to separate senescence regulation paths from the stress response because the genetic program underlying senescence is largely in line with the plant defense program. Therefore, changing one senescence parameter can also reduce plant tolerance to stress. Integrative research is needed that not only focuses on the role of single genes in the onset of senescence but also examines the conditions under which manipulation of the senescence process is beneficial for crop yield and nutritional value (a concept reviewed in Schippers et al., 2015).

This review will address the studies that allow showing (i) the survival strategy behind dark-induced senescence in barley plant and (ii) dark-induced barley leaf senescence to be used as a model, referred to in the manuscript also as DILS program, to examine leaf senescence. The idea of a program as applied to living systems has been taken from computer science. The system is built in a particular way, and so, always starts and fails in more or less the same manner.

We present transcriptomic, cytological, and physiological data that reveal events in barley DILS program, differences from developmental senescence, the time limit for dark-to-light transition for reversal of the senescence process, and progression of senescence through autophagy into the PCD phase.

**FIGURE 1:**
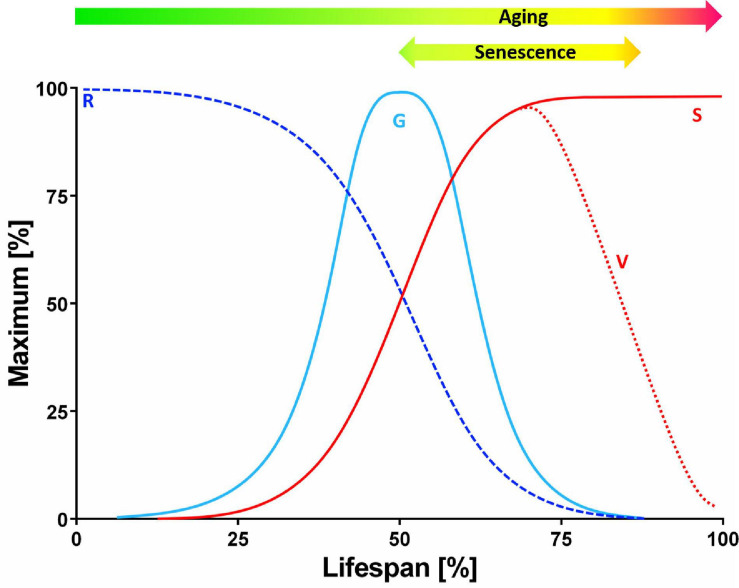
Theoretical curves of plant growth and viability. The pattern of cell mass, tissue, organ, whole plant or population growth in size (S) follows a typical sigmoidal curve. The instantaneous growth rate (G) is maximal at the point of breakdown of the S curve, whereas the relative growth rate (*R* = G/S) slopes down systematically over time. The viability of the plant (V) starts to decline gradually after the peak of G. The decreasing V is antagonistic to senescence. It should be noted that aging refers, in this context, to transition in time (not deterioration) and it comprises the period of senescence and the final phase of declining V (but it is not limited or defined by these) (modified from Thomas, 2013).

Senescence, aging, and death are topics that notoriously attract semantic disputes. The review, will begin with a brief discussion about the terminology used here. Growth in size (S) of a cell mass, tissue, organ, whole plant or population follows a typical sigmoid pattern (Figure 1). The instantaneous growth rate (G) is maximal at the point of breakdown of the S curve. The relative growth rate (*R* = G/S) decreases gradually over time. The declining life span (V) is antagonistic to senescence. Aging refers to changes (not deterioration) over time and includes, but is not limited to or defined by, the senescence period and the final phase of V decline. Senescence thus generally refers to the process or condition of growing old (from the Latin *senescere*, to grow old). Senescence, according to the current physiological understanding, is the developmental phase that: (i) constitutes an episode of transient differentiation at the termination of growth; (ii) may or may not be succeeded by death; and (iii) is completely dependent on cell viability and specific gene expression (Thomas, 2013).

## Processes in Chloroplasts Are Early Targets of Dark-Induced Barley Leaf Senescence

The first symptoms of leaf senescence are chloroplast degeneration (Dodge, 1970; Gepstein et al., 2003; Lim et al., 2007) and the decline in photosynthesis associated with it (Krieger-Liszkay et al., 2019). The earliest effects of DILS are visible in the chloroplast ultrastructure within the first 72 h of the plant’s stay in the dark. During DILS program leaf yellowing is observed, and is further identified as a result of chlorophyll (Chl) degradation, which is associated with a decrease in its autofluorescence (Sobieszczuk-Nowicka et al., 2018). Also the works of, Lichtenthaler and Grumbach (1974), Peterson and Huffaker (1975), Rolny et al. (2011), Kleber-Janke and Krupinska (1997), Scheumann et al. (1999), Scharrenberg et al. (2003), Spundova et al. (2003), and Roberts et al. (2017) on barley showed a significant decrease in chlorophyll in the early stages of dark-induced barley leaf senescence. The loss of Chl indicates the remobilization of nitrogen compounds and is accompanied by an increase in flavonoids (Flv). Most likely, this is due to the fact that, in the absence of nitrogen, excess carbon is used to synthesize polyphenols, which include Flv (Cartelat et al., 2005). Cytological studies on barley chloroplasts senescing in dark show a gradual degradation of the thylakoid system, an increase in the size and number of plastoglobuli, and, as a result, the breakdown of chloroplasts (Lichtenthaler and Grumbach, 1974; Spundova et al., 2003; Sobieszczuk-Nowicka et al., 2018). In parallel, in dark-induced senescing leaves of barley, changes in parameters determining photosynthetic quantum conversion are observed. The parameter of chlorophyll fluorescence decrease ratio (Rfd) called the vitality index ratio is dynamically falling in the early stage of DILS model in barley, which is why it is considered a significant marker parameter for stress caused by darkness. The effective quantum yield of PSII electron transport (ΦPSII) is characterized by lesser sensitivity, decreasing less intensely than Rfd (Sobieszczuk-Nowicka et al., 2018). Both Rfd and ΦPSII are referred to as net CO_2_ assimilation rates (Fracheboud and Leipner, 2003; Baker and Oxborough, 2004; Lichtenthaler et al., 2005; Wong et al., 2014). The remaining parameters slightly decreased during DILS model: maximum quantum yield of PSII of the dark-adapted state (Fv/Fm), maximum quantum yield of PSII in the light-adapted state (Fv′/Fm′), and photochemical quenching of Chl fluorescence (qP). All parameters of the values listed above showed a decrease only in the advanced stage of DILS (Sobieszczuk-Nowicka et al., 2018). A decrease in the Fv/Fm and ΦPSII parameters during senescence was also demonstrated in other studies on dark induced leaf senescence of barley (Kleber-Janke and Krupinska, 1997; Scharrenberg et al., 2003; Spundova et al., 2003).

van Doorn and Yoshimoto (2010) stated that the terminal, senescing stage of a plant cell may be reversed if the functions of the chloroplasts can be restored. To assess the limits of the ability of senescence reversal for the DILS model, it was measured the photosynthetic quantum conversion parameters and nitrogen status in barley leaves subjected to light re-exposure after various periods of dark incubation. During this process of re-exposure to light, it was observed that the parameters such as Fv/Fm, Fv′/Fm′, qP, Chl. Flv and NBI that had gradually decreased during DILS program began to recover. The Rfd and ΦPSII parameters were also restored, although with a delay. This reversal occurred in samples exposed to darkness until, but not beyond, day 7, with dark incubation lasting longer than day 7, causing an irreversible decline in all the measured parameters (Sobieszczuk-Nowicka et al., 2018). Also for each analyzed indicator, a 2-day period of light re-exposure does not suffice to return the level to that of the light control. This suggests that, in spite of the high potential of chloroplasts to restore the photochemical efficacy of solar energy conversion, the energy conversion of excitation and/or the use of potential energy, coupled with the transport of electrons, may be restricted by some unidentified factor whose reversibility is compromised.

## Rubisco Is the Largest Repository of Recoverable Nitrogen in Barley Leaf Senescing in Darkness

Mature leaves are the place of carbon (C) assimilation in the process of photosynthesis. The leaves are the sources, which means that the metabolism precursors are exported from them where they are needed: to the sinks, such as developing seeds (Lim et al., 2007; Thomas, 2013; Law et al., 2018). Sources and sinks communicate using the vascular system. At the beginning, young, expanding leaves are developing as sinks, but when they fully develop, they become sources. Leaves mainly export C obtained from photosynthesis, but when the leaves begin to grow old and cease to photosynthesize, they become sources of nitrogen (N) derived from the decomposition of leaf tissue proteins. The beginning of leaf senescence can be considered the moment of transition: from the assimilation of nutrients, to their remobilization. In other words, it is the point where leaves cease to be the sources of C, and become the sources of N (Masclaux et al., 2000; Thomas, 2013; Law et al., 2018). Therefore, leaves are, on the one hand, the location of C assimilation, and on the other, the N storage place. Behind this leaf bifunctionality is the main chloroplast protein, ribulose-1,5-bisphosphate carboxylase/oxygenase – Rubisco (Thomas, 2013). Rubisco is a photosynthetic enzyme that binds C from CO_2_ and is also a reserve of mobilizable N as Rubisco proteins contain up to 35% of total leaf nitrogen and up to 70% of chloroplasts’ nitrogen (Hörtensteiner and Feller, 2002; Krieger-Liszkay et al., 2019). Storage proteins, including Rubisco, are characterized by either a very low level, or no simultaneous protein synthesis and decomposition. When the leaves are young and growing, there is a very high level of Rubisco synthesis, and only once the synthesis stops does the protein breakdown begin (Thomas, 2013). In general, Rubisco degrades rapidly during any type of senescence (Hörtensteiner and Feller, 2002; Lan and Miao, 2019). In dark-induced senescence of barely leaves, this was confirmed by Peterson and Huffaker (1975), Simova-Stoilova et al. (2002), and Roberts et al. (2017). The level of Rubisco protein, drop significantly throughout DILS model (Sobieszczuk-Nowicka et al., 2018). This decrease was correlated with the level of expression of Rubisco large subunit gene, which decreased significantly during early phase of the process (Sobieszczuk-Nowicka et al., 2018). Also Krause et al. (1998) and Kleber-Janke and Krupinska (1997) has reported that the transcript levels of small and large subunits of Rubisco were significantly decreased during the dark-induced senescence of the barley leaves.

In contrast, the second marker protein for chloroplasts’ biochemistry – PSII reaction center D1 protein photosystems (Mattoo et al., 1989) – is quite stable and only slightly decreases in the late phase of DILS (Sobieszczuk-Nowicka et al., 2018). It might be due to the fact that the system complexes are active up to point of no return. Interestingly, the level of *PSBA* genes encoding D1 protein dropped in the early phase of DILS program. The difference between the decline in D1 protein and its transcript seems to stem from the fact that the D1 protein is under posttranscriptional control (Sobieszczuk-Nowicka et al., 2018). These differences in the degradation rate of the two chloroplast marker proteins, Rubisco and D1, in the early stage of DILS could be part of the stress adaptation strategy, as a result of which the degradation of the highly important ATP synthesis machinery in dark-induced senescence is delayed (Krupinska et al., 2012). This sequence of events is supported by the remaining microarray results of barley DILS program, which show a high level of ATP-dependent metabolism of amino acids, fatty acids, hormones and pigments, and active complexes of photosystems up to the time when senescing in darkness leaves entering the point of no return (Sobieszczuk-Nowicka et al., 2018).

## Morphological Changes of Barley Leaves Senescing in Darkness Other Than Those Described for Chloroplasts and Metabolic Modifications Associated With Them

In plant cells during dark-induced senescence chromatin condensation and nuclear fragmentation occur. At the beginning of senescence, the ultrastructure of the nucleus does not differ significantly from mature leaves. The condensation of chromatin typically starts at the periphery of the nucleus and moves inward (Sakamoto and Takami, 2014; Liu et al., 2017). During the early stages of DILS, the ultrastructure of the nucleus of barley does not differ from mature leaves. However, with the progression of senescence the shape and structure of the nucleus becomes more irregular. These changes are accompanied by DNA fragmentation (Sobieszczuk-Nowicka et al., 2018). Similar results were also reported for *Phaseolus vulgaris* (Lambert et al., 2017) and *Petroselinum crispum* (Canetti et al., 2002). The nuclear breakdown is accompanied by the release of nucleases and proteases, acidification of the cytoplasm, and rapid degradation of nucleic acids and proteins (Obara et al., 2001; Kuriyama and Fukuda, 2002), which can be a source of carbon and nitrogen. The Bnuc1 gene that encodes a BNUC1 endonuclease is generally associated with senescence (Sakamoto and Takami, 2014) and is a marker of DNA degradation of DILS. DILS program is associated with very high induction of Bnuc1 gene expression (Sobieszczuk-Nowicka et al., 2018). Wood et al. (1998) demonstrated in dark incubated barley an increase in activity of single strand preferring nuclease (SSN), which also is overexpressed in later stage of DILS.

In addition to changes in nucleus organization, changes in the tonoplast’s topology occur during dark-induced senescence. At the start of senescence, invagination of the tonoplast and cytoplasmic fragments near the vacuole can be observed. Together with these, shrinking of the protoplast is notable. With the progression of DILS in barley leaf cells, gaps in the cell membrane appear and eventually the tonoplast ruptures (Sobieszczuk-Nowicka et al., 2018). This consequently leads to the release of lytic enzymes and degradation of the nucleus and mitochondria. Usually, the rupture of the tonoplast is the final step of senescence ending in PCD (Rogers, 2015).

Since chloroplasts are one of the first to be degraded, the senescing cells must rely on mitochondria to obtain energy (Keech et al., 2007). Keech et al. (2007) reported that the morphology and abundance of these organelles change during dark-induced senescence in leaves of *Arabidopsis thaliana*. Mitochondria then are less abundant and rounder or even almost spherical. The decrease in the number of mitochondria can also be observed in different ways in different parts of the leaf, i.e., mesophyll compared to epidermal cells. Interestingly, mitochondrial numbers in stomata are not affected by dark-induced senescence (Keech et al., 2007), which takes place in developmental senescence, as was shown for *Vitis vinifera* (Ruberti et al., 2014). Also, during the final stages of the process, Keech et al. (2007) reported a cellular distribution of the organelle changes. Mitochondria clump together in loose aggregates in comparison to the relatively homogeneous distribution in control *Arabidopsis* plants (Keech et al., 2007). When the source of sugars is depleted in leaves kept in the dark, amino acids become a source of nutrients to sustain mitochondria respiration. Ammonium released in this way is assimilated by cytosolic glutamine synthetase 1 (GS1) isoforms and remobilized (Masclaux et al., 2000, 2001). The mitochondrial glutamate dehydrogenase that is known to catabolize glutamate to provide 2-oxoglutarate (2-OG) to the mitochondria is one of the catabolic enzymes releasing ammonium and 2-OG during DILS (Masclaux-Daubresse et al., 2006; Araujo et al., 2010; Kleessen et al., 2012). During DILS in barley one can also observe up-regulation of other enzymes responsible for remobilization of degraded nitrogen compounds such as cysteine and aspartyl proteases, ubiquitination enzymes, Hsp70, cytosolic Gln synthetase (Gln-1-3 isoform of low affinity to ammonia), and Orn cycle enzymes (P5C dehydrogenase, arginase, acetyl-Orn transaminase) (Sobieszczuk-Nowicka et al., 2018). Also during this specific time, lipid catabolism increases, which suggests that lipid degradation may participate in the production of energy, which in turn involves succinate synthesis within glyoxysomes and export thereafter to mitochondria (Sobieszczuk-Nowicka et al., 2018). Mitochondrial metabolism during DILS in barley is demonstrated to be important in relocation of recycled carbon and nitrogen substrates which come from proteins, lipids and other cellular components, since after crossing the point of no return the deterioration processes coupled with respiration intensify, which is observed by e.g., overexpression of genes involved in vesicle recycling (signalosome complex, SNARE complex or vesicle-fusing ATPase) (Sobieszczuk-Nowicka et al., 2018). Increased expression of key genes of gluconeogenesis and glycolysis along with upregulation of glyoxysomal enzymes are also supportive of recycling of substrates (Hollmann et al., 2014).

## Autophagy and Senescence Occur Synchronously in Dark-Induced Barley Leaf Senescence

During plant growth and development, damaged or aging cells’ components undergo degrading processes inside vacuoles in a process called autophagy. Autophagy does not occur at a very high level under physiological conditions, and it is a housekeeping process during normal conditions, allowing the organism to adapt to changing environmental conditions and allowing its survival and prolonging its life span (Avila-Ospina et al., 2014; Borek et al., 2017; Stefaniak et al., 2020). The process of autophagy is phylogenetically conserved, involving intracellular degradation where cytoplasmic compounds are broken down in the vacuole to supply basic components and energy to maintain essential functions. During autophagy also damaged cells and toxic compounds are utilized (van Doorn and Woltering, 2005; Janawad et al., 2012). As DILS is a process of transition from nutrient assimilation to nutrient remobilization (Buchanan-Wollaston et al., 2005), autophagy plays a key role in it. In plants, several types of autophagy can be distinguished, mainly microautophagy and macroautophagy (Bassham et al., 2006) as well as a third, plant-specific pathway, called megaautophagy (Floyd et al., 2015). Microautophagy consists of tonoplast invagination which results in engulfment of tonoplast and cytoplasmic components by intravacuolar vesicles and their uptake into the vacuole (Bassham et al., 2006; Sienko et al., 2020). Megaautophagy also leads to disintegration of cell contents using vacuolar enzymes, but the material is not taken up inside the vacuole. Instead, vacuolar hydrolases are released to the cytoplasm after permeabilization and disruption of the tonoplast (Hara-Nishimura et al., 2005; Bollhöner et al., 2012). In turn, macroautophagy begins with the appearance in the cytoplasm of an elongated vesicle (phagophore) composed of a single, bilayer lipid-protein membrane, which elongates and engulfs a fragment of the cytoplasm with cell organelles and/or protein complexes. The resultant autophagosome is then transported to the vacuole, where the cargo is hydrolyzed (Borek et al., 2015). Macroautophagy is responsible for maintaining standard function of the cell (Lamb et al., 2013). Hence, organized development of DILS requires effective recycling machinery which allows the correct development of a plant to be maintained. During dark-induced senescence, the increased degradation of macromolecules such as nucleic acids, proteins, and sugars, provides components for regulated recycling and reuse by other parts of the plant (Jing et al., 2003).

It was reported that Rubisco and its degradation products can be transported into vacuoles via Rubisco-containing bodies (RCBs) after darkness treatment (Chiba et al., 2003). Ishida et al. (2008) found that RCB targeting to the vacuole are autophagy-dependent. They also observed, using plants expressing both the GFP–ATG8 fusion marker specific for autophagosomes and autophagic bodies, and stroma-targeted RFP, co-localization of the two fluorescent markers within the vacuole (Ishida and Yoshimoto, 2008; Ishida et al., 2008).

**FIGURE 2:**
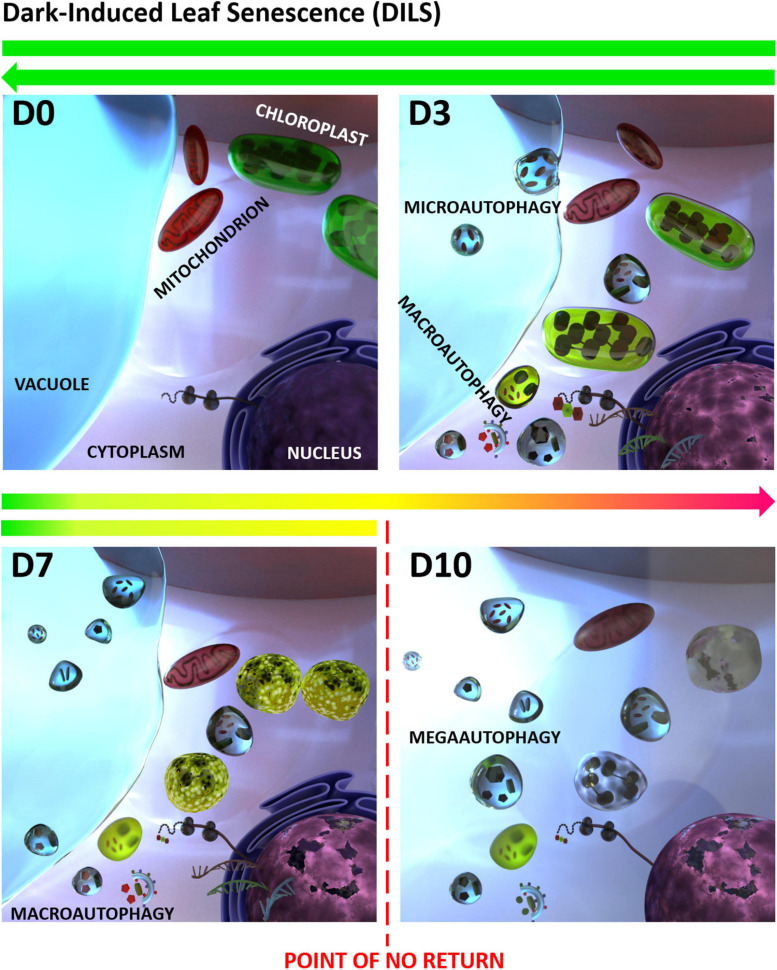
Dark-induced leaf senescence (DILS) model vs. autophagy. D0 stands for control, D3 to D10 stand for days of senescence. A critical time limit was identified when it is possible to reverse the leaf senescence and prevent cell death. It was found that in DILS autophagy participates in the circulation of cell components and acts as a quality control mechanism during senescence. DILS is also a good model for studying the pathways of autophagy and programmed cell death. At each stage, DILS is accompanied by different types of autophagy: micro-, macro-, and mega-autophagy. Despite the progression of macroautophagy, the symptoms of degradation can be reversed. How the function of autophagy switches between cell survival and cell death is not known (modified from Sobieszczuk-Nowicka et al., 2018).

In leaves senescing in darkness autophagy is apparent as small autophagic bodies in vacuoles, presence of autophagosomes in protoplasts and in the process of tonoplast rupturing. Also, a number of autophagy-related genes (ATGs) have been identified during dark-induced senescence, which are highly expressed during the progress of the process (Chung et al., 2009; Xia et al., 2012; Avila-Ospina et al., 2016; Sobieszczuk-Nowicka et al., 2018). In barley Sobieszczuk-Nowicka et al. (2018) showed that at the onset of DILS program the tonoplast membrane invaginates, small cytoplasmic fragments are near the vacuole and the cytoplasm shrinks, which indicates the involvement of microautophagy in the early stages of senescence. When DILS proceeds, leaf cells demonstrate cell membrane discontinuity, which indicates the processes of macroautophagy. However, even at this stage, the effects of DILS degradation processes have been shown to be reversible. In the final stages of DILS these processes are followed by megaautophagy, which is a rupture of the tonoplast and release of hydrolases. In consequence, organelles undergo progressive degradation and are localized in the center of the cell, and the intracellular compartmentation is lost due to the plasma membrane loosening. When megaautophagy occurs, the cell enters the “point of no return” after which degradation of the cell nucleus and mitochondria take place, and the cell proceeds into PCD, Sobieszczuk-Nowicka et al. (2018). Figure 2 summarizes the described stages of senescence occurring in barley cells during darkness, taking into account the characteristic autophagy types: micro-, macro-, and mega-autophagy. Despite the progression of macroautophagy, the symptoms of degradation can be reversed, until megaautophagy occurs, showing a clear point of no return. Together with these changes a number of *ATGs* are upregulated as well as genes encoding vacuolar-processing enzymes (i.e., α*VPE* and *VPE2c*), whose expression increases with the progression of senescence. *VPE* genes are involved in ATG-independent alternative cell degradation pathways via senescence-associated vacuole formation. During dark-induced senescence a vital role is played by these 0.5–0.8 μm vacuoles (SAVs). SAVs have been identified in the senescent leaves of several plants, including soybean, *Arabidopsis*, and tobacco, but are absent in non-senescing leaves (Martinez et al., 2008a,b). There are soluble proteins (such as Rubisco) and resident proteases (such as senescence-specific *SAG12*) in the acid lumen of these vacuoles (Pascual et al., 1994; Otegui et al., 2005; Martinez et al., 2008a,b). In DILS relative to the expression of α*VPE* and *VPE2c*, that of the known senescence-activated marker gene Cys protease (*SAG12*) was minimally induced (Sobieszczuk-Nowicka et al., 2018).

Turnover of macromolecules via selective autophagy may contribute to cell homeostasis, nutrient recycling, and clearance of damaged structures during DILS. The fact that autophagy might be important in N mobilization in the course of developmental leaf senescence of barley has been proposed before (Hollmann et al., 2014). We do not know the mechanisms that condition the metabolic reprogramming that directs to or leads out leaf cells senescing in darkness from the PCD pathway, switching cells between survival and death. However, we know that cell death occurs by suppressing macroautophagy and triggering megaautophagy. It is possible that VPEs are mediators of the crosstalk between senescence-dependent autophagy and PCD (Patel et al., 2006; Floyd et al., 2015; Wang et al., 2018). Supporting the hypothesis that autophagy works to keep cells healthy, controlling the cell component turnover during dark-induced senescence, is the fact that plants with low autophagic activity, i.e., *Arabidopsis* mutants, are more susceptible to stress and exhibit premature senescence symptoms and cell death (Phillips et al., 2008). The efficiency of regulation of autophagic processes is a symptom of the vitality of senescing cells, which at each stage must hold the ability to maintain homeostasis. Thus, we suggest that a critical step that determines the point of no return in DILS model is macroautophagy control.

## Dark-Induced Barley Leaf Senescence Versus Developmental Barley Leaf Senescence; Transcriptomic Study

Large scale data analysis of barley DILS program using microarrays and re-analyzed data of Christiansen and Gregersen (2014) and Hollmann et al. (2014) of developmental leaf senescence showed that genes expressed during DILS and developmental senescence show quite some similarities (Sobieszczuk-Nowicka et al., 2018). Genes encoding glyoxysomal citrate synthase (Hollmann et al., 2014) and mitochondrial succinate dehydrogenase (Christiansen and Gregersen, 2014) are common for both types of senescence and are upregulated during that time (Christiansen and Gregersen, 2014; Sobieszczuk-Nowicka et al., 2018). The regulation of these processes that allow for the organelles to gain energy seems to be necessary for the cell to replace the lack of ATP synthesis in chloroplasts for both dark-induced senescence and developmental senescence as the chloroplasts are dismantled at early stages of senescence, whereas mitochondria prevail until cell death (Peterson and Huffaker, 1975; Matile, 1992; Chrobok et al., 2016). Another resemblance is the downregulation of malate dehydrogenase, which is correlated with inhibition of glyceraldehyde-3-phosphate dehydrogenase in chloroplasts (Sobieszczuk-Nowicka et al., 2018). This enzyme functions as a fragment of the starch-degradation pathway that supports malate for other organelles in unstressed cells (Foyer et al., 2011). In both senescence models, low-affinity ammonia remobilization (by a cytosolic isoform of Gln synthetase 1 and Glu dehydrogenase) and Orn cycle transamination are also found to be activated (Sobieszczuk-Nowicka et al., 2018). Another similarity between DILS and developmental senescence of barley is common expression of some cysteine proteases (CPs). They are the most abundant enzymes associated with leaf senescence (Bhalerao et al., 2003; Parrott et al., 2010). Scharrenberg et al. (2003) have also reported that transcripts of CP *HvSF42* (HvPap-1) increase in both senescence scenarios in barley. What is more, these authors demonstrated, that in both dark-induced and developmental senescence an *NAC* transcription factor (HvSF6/HvNAC008) is up-regulated (Scharrenberg et al., 2003). *HvSF6* was observed to be induced by the cross talk of jasmonic acid and ethylene in senescing barley in both developmental and dark-induced senescence (Scharrenberg et al., 2003). This observation was later confirmed by Christiansen et al. (2011), who identified 48 NACs in barley. This suggests the participation of *NAC* transcription factors as regulators of a range of processes in plant development and stress responses, senescence being one of them.

On the other hand, the most notable differences in gene medleys between DILS and developmental senescence were observed for signaling pathways which are activated by plant hormones, lipid catabolism, low-affinity ammonia remobilization, carbohydrate metabolism and DNA and RNA methylation. The differences between DILS and developmental senescence in the activity of carbohydrate and lipid metabolism enzymes were proven by recording the increase in expression of glycolytic glyceraldehyde-3-phosphate dehydrogenase and enolase in the first process but not in the other, whereas during DLS β-amylase and trehalose-6-phosphate synthase are up-regulated (Sobieszczuk-Nowicka et al., 2018). Similar results were obtained in *Arabidopsis*, in which lipid catabolism genes are considerably more upregulated during dark-induced senescence than in developmental (Buchanan-Wollaston et al., 2005). This causes an increase in beta-oxidation due to depletion of carbohydrates. In barley DILS there was noted upregulation of gluconeogenesis, to which the by-product of this pathway, phosphoenolpyruvate, can be directed. The pivotal enzyme of this process, pyruvate phosphate dikinase, is downregulated during developmental senescence. This is consistent with an increase in expression of Suc synthase in DILS, which was decreased during developmental senescence (Sobieszczuk-Nowicka et al., 2018). A number of other crucial differences have also been revealed between these two processes. In DILS downregulation of C- and D-type phospholipase genes can be observed. Phospholipases are well-established enzymes taking part in both lipid catabolism and signaling pathways dependent on GTP, where they constrain the α-subunit of G-protein coupled receptors (Fukami, 2002; Jenkins and Frohman, 2005). Another process differing between DILS and developmental senescence is the gibberellin synthesis pathway, which is upregulated in the former but not in the latter. But then, signaling through jasmonic acid and auxin seems to be crucial for developmental senescence as the overexpression of 3-ketoacyl-CoA thiolase and auxin response factor 19 is observed during this process. On the other hand, in *A. thaliana* during dark-induced senescence, three cytosolic glutamine synthetase genes are not expressed, in contrast to developmental senescence (Buchanan-Wollaston et al., 2005). The mentioned enzymes have a pivotal role in N mobilization, which may imply that different pathways may operate during differentially triggered senescence. The upregulated genes also observed only in DILS in barley are ones encoding aminotransferases (Sobieszczuk-Nowicka et al., 2018). The release of branched-chain amino acids confirms this upregulation during dark incubation of *Arabidopsis* leaves, which counteracts the toxicity of free ammonia from amino acids with a high N:C ratio (Law et al., 2018). Interestingly, genes responsible for proteolysis are differentially expressed in DILS and developmental senescence. During DILS in barley, for example, ubiquitin-conjugated enzyme is down-regulated while during development this enzyme is overexpressed. Similarly, an opposite expression profile can be observed for a vacuolar-processing enzyme precursor (changes in *VPE* are discussed in more detail in the section on autophagy), which is overexpressed during DILS whereas in developmental senescence it is down-regulated (Sobieszczuk-Nowicka et al., 2018). Also some members of subtilases – a family of serine-rich proteases (SPs) – can be differentially expressed in barley depending on the type of senescence. Subtilases in DILS, denoted *HvSBT45*, compared to developmental senescence were down-regulated during late stages of DILS, while in developmental senescence their expression level rises (Christiansen and Gregersen, 2014; Sobieszczuk-Nowicka et al., 2018). Roberts et al. (2017) tested gene expression of eleven subtilases in barley plants subjected to developmental and dark-induced senescence. They demonstrated that two out of all tested enzymes (*HvSBT3* and *HvSBT6*) are up-regulated in both senescence conditions, while one differentiated developmental and dark-induced senescence. *HvSBT2* is only up-regulated during dark-induced senescence (Roberts et al., 2017). This shows that different subgroups of proteases take part in proteolysis, depending on the type of factor inducing senescence. Lastly, the RNA methylation index is higher in DILS than in developmental senescence, showing an increase in gene expression of for example RNA 2-O-methyltransferase fibrillarin two during DILS and inhibition during developmental senescence (Sobieszczuk-Nowicka et al., 2018; Ostrowska-Mazurek et al., 2020).

## The Dark-Induced Leaf Senescence Crop Model and Its Point of No Return – a Summary

There has been developed a crop model that demonstrates and explains early and late events of DILS and identifies the time limit for dark to light transition for reversal of the induced-senescence process within a leaf – DILS. DILS in barley occurs in two phases. The first phase is more strongly emphasized by cessation of photosynthesis, loss of chlorophyll, and disintegration of chloroplasts. Disintegration of chloroplasts correlated with the degradation of Rubisco and PsbA-D1 proteins. Despite the advanced state of macroautophagy in this phase, the processes of degradation turned out to be reversible. The reversal of DILS program involves regaining photosynthesis and increase of chlorophyll content, and it takes place irrespectively of the activation of ATG genes. The second, terminal phase, occurring beyond day 7 of darkness, is characterized by irreversibility of senescence and its progression into PCD, exemplified by the involvement of both autophagy and PCD pathways, and involves disruption of the nucleus, mitochondria, chromatin condensation accompanied with nDNA fragmentation, shrinking of the protoplast, tonoplast interruption, and disintegration of the cell membrane.

Non-invasive methods for quantifying photosynthetic efficiency and barley leaf nitrogen status established the time frame during which DILS enters the irreversible phase. Rfd is determined there as the earliest parameter that correlated well with the cessation of photosynthesis, together with the appearance of micro-autophagy symptoms. DILS program is also found to be characterized by the upregulation of processes that enable the recycling of degraded metabolites in darkness, including increased ${\text{NH}}_{4}^{+}$ remobilization, gluconeogenesis, glycolysis, and partial upregulation of glyoxylate and tricarboxylate acid cycles.

## Author Contributions

ES-N conceived the topic of the manuscript. EP-L, ES, and ES-N wrote the manuscript. EP-L prepared the figures. ES was responsible for the layout of the manuscript and prepared the table. ES-N coordinated writing of the manuscript. All authors listed have made a substantial, direct and intellectual contribution to the work, and approved it for publication.

## Conflict of Interest

The authors declare that the research was conducted in the absence of any commercial or financial relationships that could be construed as a potential conflict of interest.
